# Clinical next generation sequencing to identify actionable aberrations in a phase I program

**DOI:** 10.18632/oncotarget.4040

**Published:** 2015-05-08

**Authors:** Genevieve M. Boland, Sarina A. Piha-Paul, Vivek Subbiah, Mark Routbort, Shelley M. Herbrich, Keith Baggerly, Keyur P. Patel, Lauren Brusco, Chacha Horombe, Aung Naing, Siqing Fu, David S. Hong, Filip Janku, Amber Johnson, Russell Broaddus, Raja Luthra, Kenna Shaw, John Mendelsohn, Gordon B. Mills, Funda Meric-Bernstam

**Affiliations:** ^1^ Division of Surgical Oncology, Department of Surgery, Massachusetts General Hospital, Harvard Medical School, Boston, MA, USA; ^2^ Department of Surgical Oncology, The University of Texas MD Anderson Cancer Center, Houston, TX, USA; ^3^ Department of Investigational Cancer Therapeutics, The University of Texas MD Anderson Cancer Center, Houston, TX, USA; ^4^ Department of Hematopathology, The University of Texas MD Anderson Cancer Center, Houston, TX, USA; ^5^ Department of Gynecologic Oncology and Reproductive Medicine, The University of Texas MD Anderson Cancer Center, Houston, TX, USA; ^6^ Department of Bioinformatics and Computational Biology, The University of Texas MD Anderson Cancer Center, Houston, TX, USA; ^7^ Department of Sheikh Khalifa Bin Zayed Al Nahyan Institute for Personalized Cancer Therapy, The University of Texas MD Anderson Cancer Center, Houston, TX, USA; ^8^ Department of Pathology, The University of Texas MD Anderson Cancer Center, Houston, TX, USA; ^9^ Department of Systems Biology, The University of Texas MD Anderson Cancer Center, Houston, TX, USA

**Keywords:** genomic sequencing, actionable genes

## Abstract

**Purpose:**

We determined the frequency of recurrent hotspot mutations in 46 cancer-related genes across tumor histologies in patients with advanced cancer.

**Methods:**

We reviewed data from 500 consecutive patients who underwent genomic profiling on an IRB-approved prospective clinical protocol in the Phase I program at the MD Anderson Cancer Center. Archival tumor DNA was tested for 740 hotspot mutations in 46 genes (Ampli-Seq Cancer Panel; Life Technologies, CA).

**Results:**

Of the 500 patients, 362 had at least one reported mutation/variant. The most common likely somatic mutations were within *TP53* (36%), *KRAS* (11%), and *PIK3CA* (9%) genes. Sarcoma (20%) and kidney (30%) had the lowest proportion of likely somatic mutations detected, while pancreas (100%), colorectal (89%), melanoma (86%), and endometrial (75%) had the highest. There was high concordance in 62 patients with paired primary tumors and metastases analyzed. 151 (30%) patients had alterations in potentially actionable genes. 37 tumor types were enrolled; both rare actionable mutations in common tumor types and actionable mutations in rare tumor types were identified.

**Conclusion:**

Multiplex testing in the CLIA environment facilitates genomic characterization across multiple tumor lineages and identification of novel opportunities for genotype-driven trials.

## INTRODUCTION

The use of molecular profiling in cancer research and clinical care has begun to rapidly evolve due to changes in available technology, applicability to patient samples, and decreases in the cost of sequencing technologies. Given the resources now available, there has been an increasing interest in creating personalized medicine platforms to allow selective assessment of a given patient's tumor in real time, with the ability to utilize that information for patient care decisions. International profiling efforts such as The Cancer Genome Atlas Project (TCGA) [1] and the International Cancer Genome Consortium have begun to establish the baseline mutational profile of various cancers [2], but the application of this molecular knowledge in the care of individual patients has yet to be fully realized. Furthermore the population of patients and tumors analyzed in these international efforts frequently does not reflect those likely to enter clinical trials. At the University of Texas MD Anderson (MD Anderson), the Institute for Personalized Cancer Therapy (IPCT) has established protocols for molecular profiling of patient tumors with the goal of establishing personalized medicine platforms for individualized cancer care. Other groups have undertaken pilot studies of this type [3], although thus far the overall number of cancer patients enrolled onto these types of protocols has been small, predominantly due to the need for significant institutional support for large-scale endeavors of this type and the lack of adequate numbers of clinical trials with targeted agents. Thus the spectrum of aberrations seen in patients likely to enter clinical trials both within and across diseases remains only partially realized.

The utility of pairing the molecular profiling of tumors and the application of targeted therapies is exemplified by the success of this approach in Her2-positive breast cancer [4] and *BRAF V600E* mutated melanoma [5] amongst others. Although improvements in outcomes have been demonstrated with the selective application of therapies to an identified cohort of likely benefit, even within a given cancer type, there is a distribution of activating mutations amongst patients that creates heterogeneity of the patient population and impacts eligibility for treatment with targeted agents. The use of a multi-gene screening panel may potentially allow a more personalized approach to cancer therapy by identifying less common but potentially actionable mutations across diseases as well as rare mutations within diseases. However, the relationship between molecular targeting, the intrinsic gene expression pattern in the lineage, the presence of co-mutations and the tumor microenvironment is complicated and actionability of mutations may vary from one tissue and one patient to another.

We examined the first 500 patients with advanced cancer and sequenced on the IPCT molecular profiling study in the Department of Investigational Cancer Therapeutics (the Phase I Program) at MD Anderson, a population of patients representing multiple disease sites, in order to compare the utility and findings of a molecular profiling platform in a heterogeneous patient population with advanced cancer likely to enter clinical trials.

## RESULTS

The first 500 patients sequenced on this Institutional Review Board (IRB)-approved protocol in the Department of Investigational Cancer Therapeutics were analyzed. In the same time periods 117 patients consented to the study but did not undergo sequencing due to lack of available archival tissue or inadequate quantity DNA. Almost all patients had archival tissue used for analysis (surgical samples or core biopsies in FFPE), median time from the original sample acquisition was 582 days. Nearly two thirds had primary tumors analyzed. The median time from study consent to return of genomic sequencing data was 17 days.

**Figure 1:**
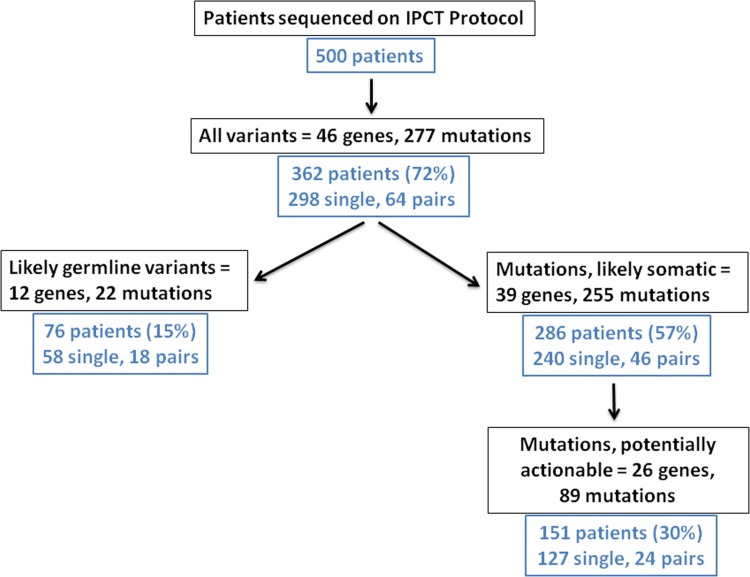
Flowchart of first 500 patients sequenced on a genomic profiling protocol in the Department of Investigational Cancer Therapeutics Of the first 500 patients enrolled on the IPCT protocol, 362 (72%) were found to have variants on the 46-gene panel screening. Of these 74 patients (15%) had likely germline variants, which were excluded for subsequent analysis. The following 286 patients (57%) had likely somatic mutations found in 39 genes. Of these, 151 patients (30%) had potentially actionable mutations in 26 genes.

Of 500 patients who underwent genomic sequencing 362 patients (72%) had at least one alteration detected on this 46 gene panel (Figure 1 and Table 1). Of the mutations detected, 298 were from single patient samples, while 64 patients had paired primary and metastatic samples available for analysis. Amongst the mutations found, 22 mutations in 12 genes were classified as likely germline variants as determined by definitions described in Methods. The most common germline variants found were in *KIT* (17%), *MET* (6%), *ATM* (5%), *KDR* (5%), and *TP53* (2%) with variable representation in other genes. The remaining mutations were designated as “likely somatic” and will be referred to as somatic in the subsequent text. Based on this definition, somatic mutations were detected in 286 out of a total of 500 patients (57%) representing 240 single patient samples and 46 patients with paired primary and metastasis. A gene was considered actionable if its product can be targeted with an approved or investigational therapy either directly or indirectly targeted (by targeting a downstream pathway [9]). The definition of actionable was not tissue-specific and may not reflect therapeutic efficacy or availability across cancer types. We next identified 26 genes with actionable targets, which we designated as potentially actionable and found 89 actionable mutations present in 151 patients (30%) representing 127 single patient samples and 24 primary/metastasis pairs.

**Figure 2:**
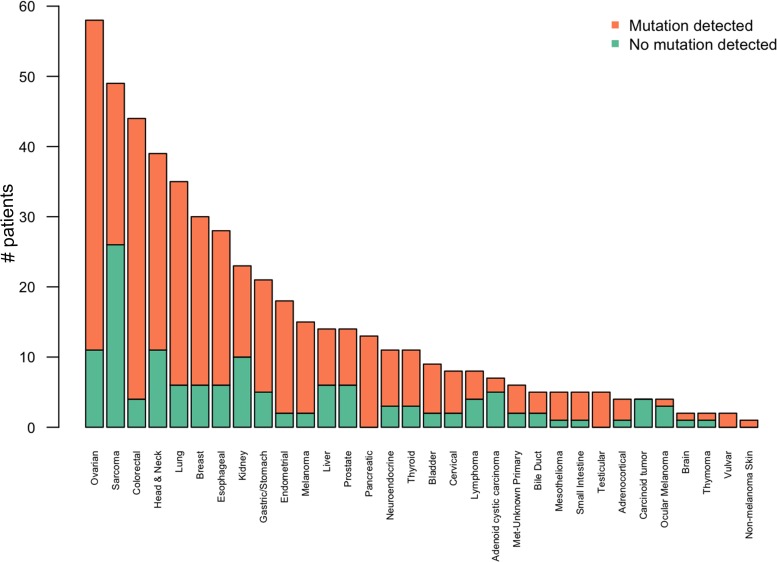
Number of patients with likely somatic mutations seen by tumor type The most common tumor profiled was ovarian, followed by sarcoma, colorectal and lung cancer. However, the distribution of tumors with mutations detected varied by tumor type. For example, despite a large number of sarcoma patients within this population (*n* = 49), the proportion of likely somatic mutations detected in sarcoma patients was relatively low (20%).

**Figure 3:**
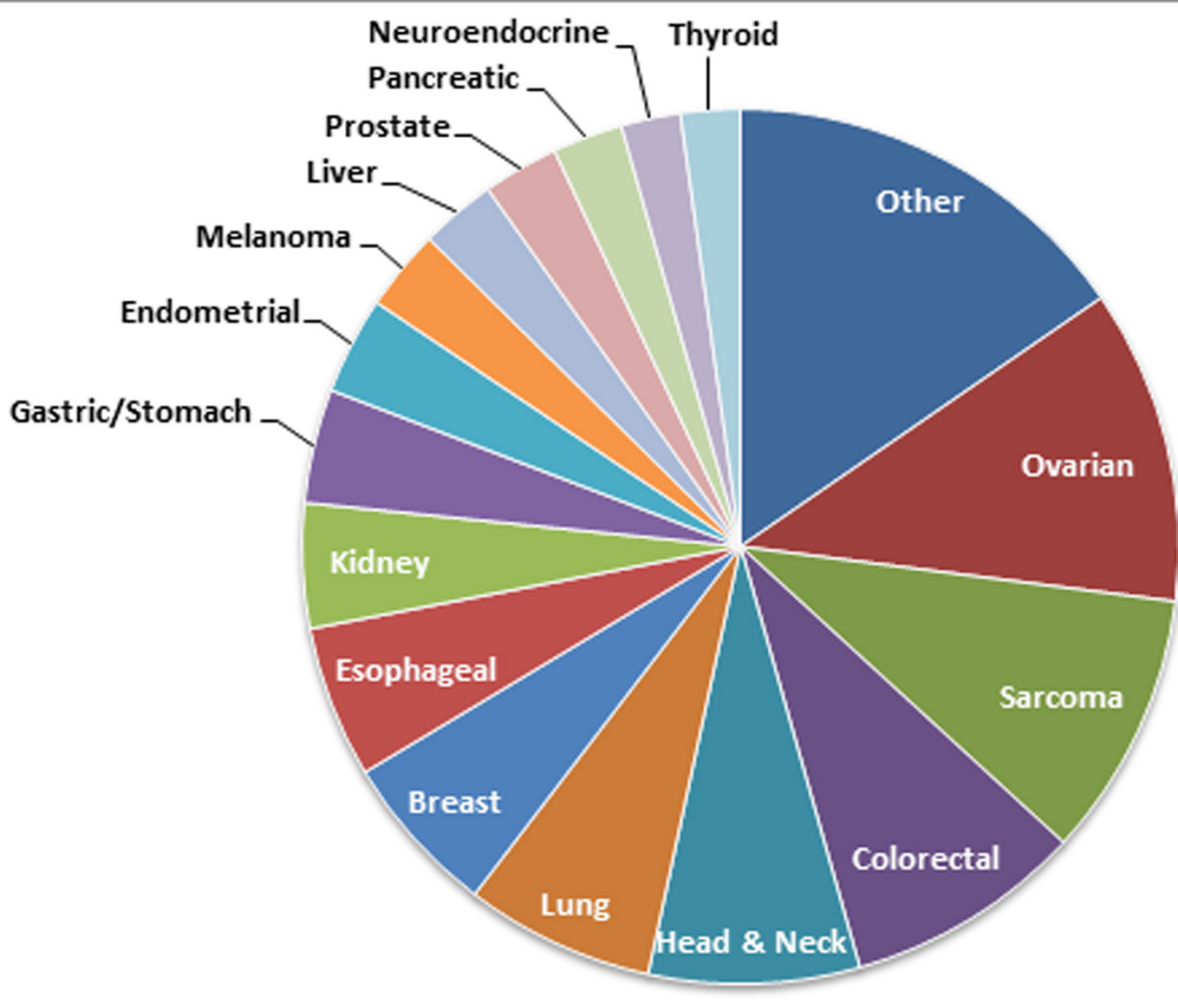
Relative distribution of samples across tissue types (*n* > 10) Only tumor types with more than 10 patients were analyzed in the subsequent analysis, and the relative proportion of those tumor types can be seen in this pie chart. The absolute numbers are as follows: ovarian (*n* = 58), sarcoma (*n* = 49), colorectal (*n* = 44), lung (*n* = 35), head & neck (*n* = 35), breast (*n* = 30), esophageal (*n* = 28), kidney (*n* = 23), gastric (*n* = 21), melanoma (*n* = 15), prostate (*n* = 14), liver (*n* = 14), pancreatic (*n* = 13), endometrial (*n* = 18), neuroendocrine (*n* = 11), and thyroid (*n* = 11) and other (*n* = 77).

**Figure 4:**
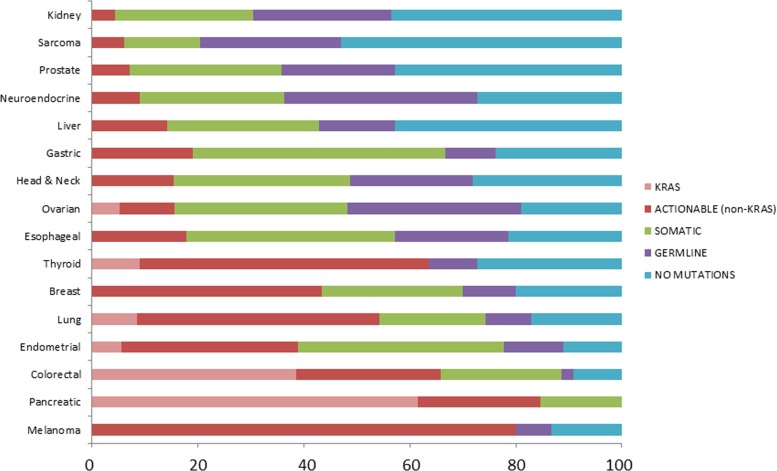
Percentage of patients with type of variants seen by tumor type The data was reviewed according to tumor type a)nd demonstrated great variation in the proportion of *KRAS*, potentially actionable somatic mutations excluding *KRAS*, non-actionable somatic, likely germline variants, and absence of mutations found using our 46 gene panel. While sarcoma has the fewest number of mutations/variants detected, pancreatic tumors had no germline variants found and melanoma had the largest proportion of potentially actionable mutations detected. Many of these findings may represent the inherent selection bias of mutations represented on the gene panel, which has been focused on identifying known actionable mutations with potential therapeutic targets.

**Figure 5:**
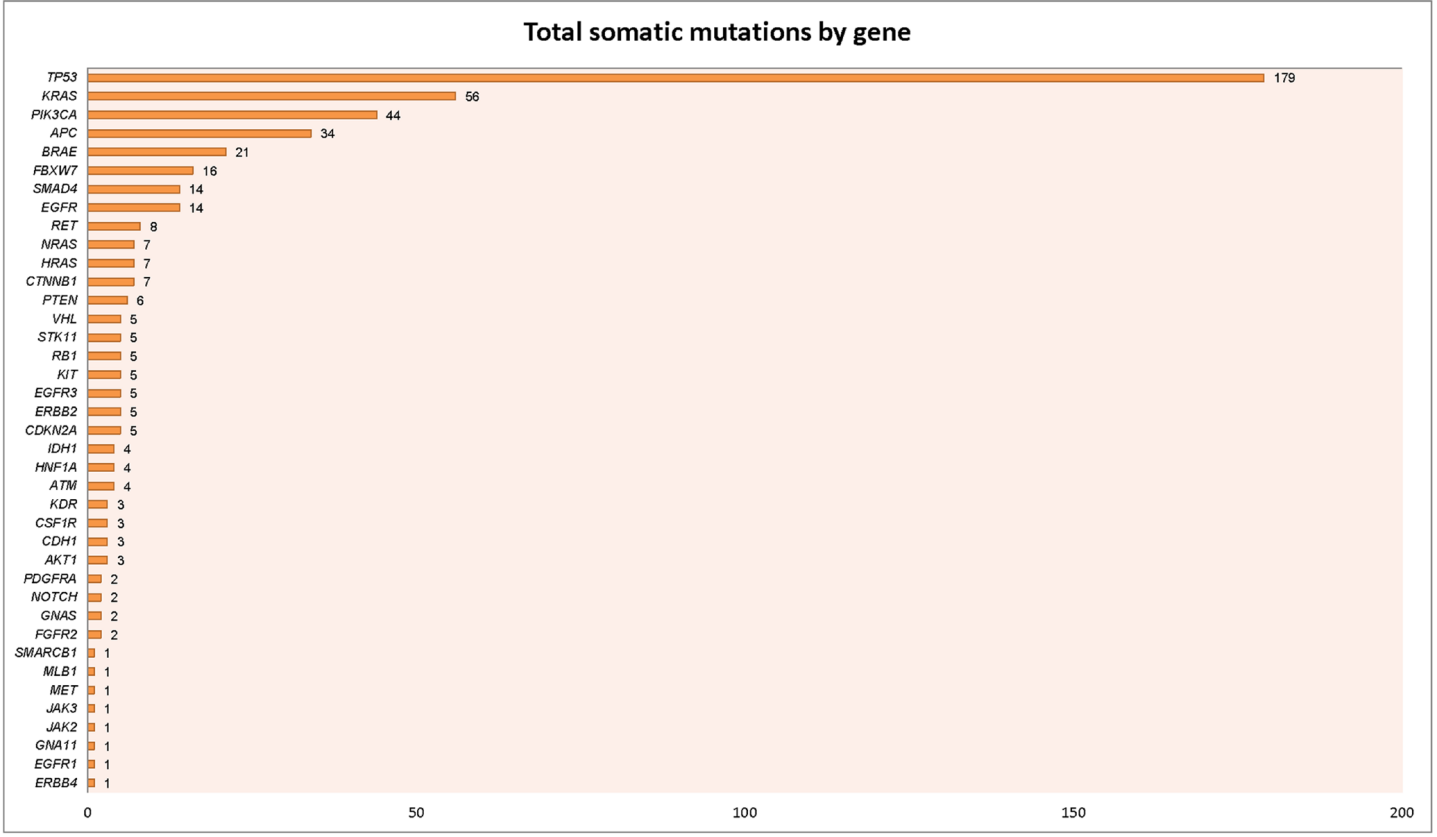
Number of mutations by gene **A.** Likely somatic mutations are displayed according to the gene involved, with mutations in *TP53* being most common (*n* = 179), followed by *KRAS* (*n* = 56) and *PIK3CA* (*n* = 44). The median number of somatic mutations per gene was 1. **B.** A subset of these genes, identified as potentially actionable mutations are listed according to the gene involved, with *KRAS* being the most common actionable mutation (*n* = 56), followed by *PIK3CA* (*n* = 44), and *BRAF* (*n* = 21).

Our initial approach was to examine mutations across all tumor types represented to establish an overview of the mutational landscape across diseases (Figure 2). Subsequently, we elected to look in more detail at mutation frequencies in tumors with greater patient representation (*n* ≥ 10 samples). The distribution of these tumor types can be seen in Figure 3. The most common tumors submitted for analyses were ovarian (*n* = 52), sarcoma (*n* = 49), and colorectal (*n* = 44). Proportionally pancreatic tumors had the largest number of mutations detected (100% overall, 100% somatic, 85% potentially actionable, noting that *KRAS* was designated potentially actionable in this analysis). This was followed by melanoma (93% overall, 86% somatic, 86% actionable) and colorectal cancer (91% overall, 89% somatic, 66% actionable). On the other end of the spectrum, sarcoma had the lowest proportion of mutations detected on this panel (47% overall, 20% somatic, 8% actionable) (Figure 4 and Table 1). Frequency of *KRAS* mutations and actionable mutations excluding KRAS by tumor type is demonstrated in this figure. Looking across disease types at the overall incidence of somatic mutations (Figure 5) demonstrates that *TP53* was the most commonly mutated gene followed by *KRAS*, and *PIK3CA*. However, when looking exclusively at the proportion of mutations in potentially actionable genes, *KRAS*, *PIK3CA*, *BRAF*, and *EGFR* were the most commonly detected actionable mutations.

**Table 1:** CMS46 hotspot mutation/variants in tumor types (n > 10 pts)

Tumor Type	Mutation (%)	Somatic (%)	Actionable (%)	Actionable w/o KRAS (%)	Total Pt #
Ovarian	81	48	16	10	58
Sarcoma	47	20	6	6	49
Colorectal	91	89	66	27	44
Lung	83	74	54	46	35
Head & Neck	72	49	15	15	39
Breast	80	70	43	43	30
Esophageal	79	57	18	18	28
Kidney	57	30	4	4	23
Gastric	76	67	19	19	21
Melanoma	87	80	80	80	15
Prostate	57	36	7	7	14
Liver	57	43	14	14	14
Pancreatic	100	100	85	23	13
Endometrial	89	78	39	33	18
Neuroendocrine	72	26	9	9	11
Thyroid	73	64	64	55	11

Breaking down the data according to cancer type, it becomes clear that melanoma has the highest proportion of potentially actionable mutations (mostly *BRAF*), followed by pancreatic cancer (*KRAS*) and colorectal cancer (*KRAS* and *PIK3CA*) as seen in Figure 5. It is notable that although our study population is limited to patients who had multiplex testing after being referred to the Department of Investigational Cancer Therapeutics, it is possible that selected patients already had single gene testing and had identified mutations (e.g. *BRAF* mutations), thus enriching for populations with actionable targets in certain diseases.

**Figure 6:**
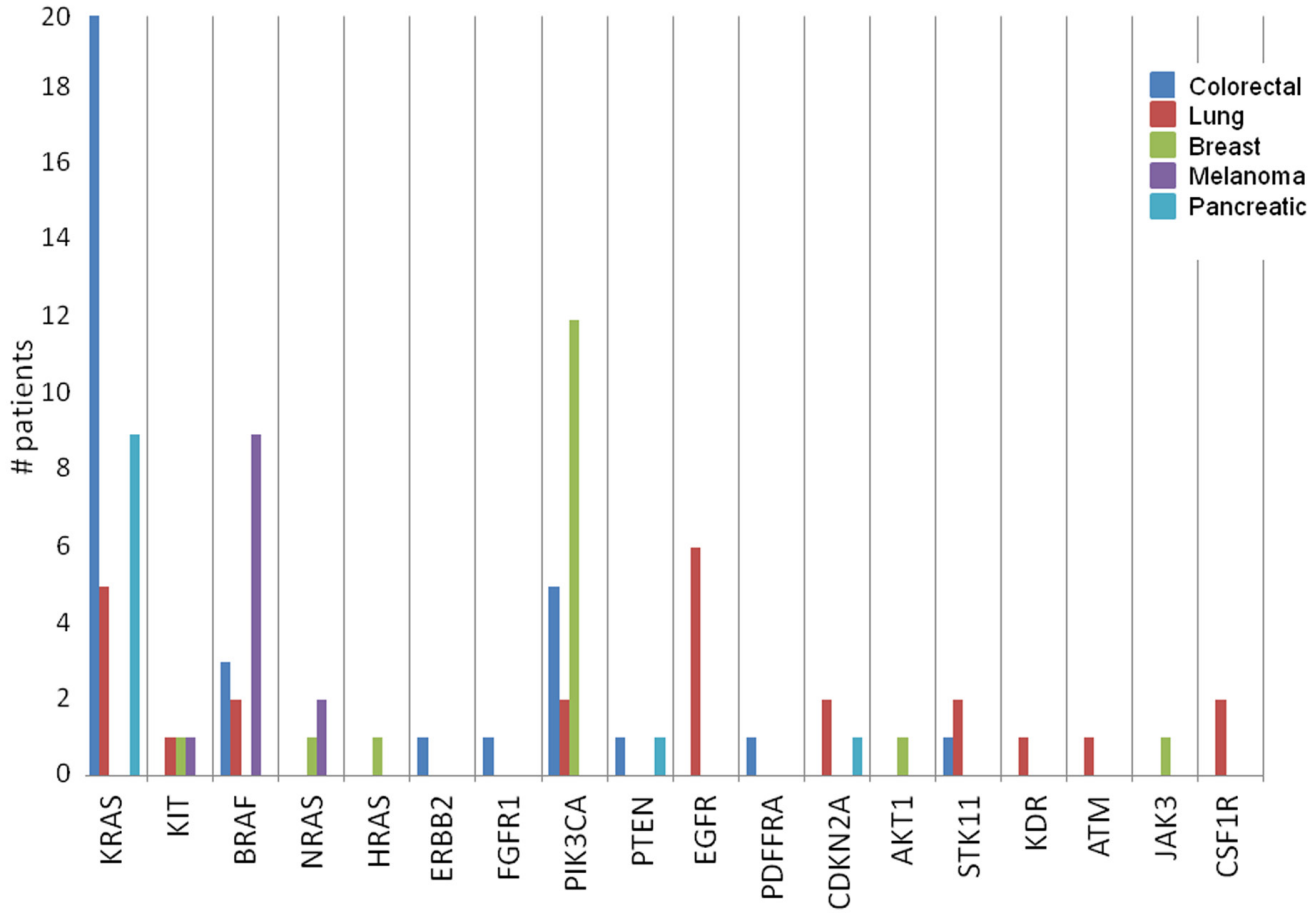
Frequency of potentially actionable mutations by cancer type This figure focuses exclusively on potentially actionable mutations, stratifying the gene mutations according to tumor type to demonstrate the relative abundance of these mutations across the various cancers included in this study. *KRAS* mutations are most commonly seen in pancreatic and colorectal tumor specimens, *BRAF* mutations are most commonly found in melanoma samples, and *PIK3CA* mutations are most commonly detected in breast cancer samples as you would expect. However, there are a smaller proportion of actionable mutations found in tumors less commonly associated with these mutations, such as *NRAS* mutations in breast cancer and *PIK3CA* mutations in lung cancer.

Next, we examined the proportion of potentially actionable mutations within the various cancer types present in our patient population (Figure 6). Mutations in *KRAS* were most common in pancreatic cancer followed by colorectal cancer. *BRAF* and *NRAS* mutations were most common in melanoma. *PIK3CA* mutations were most common in breast cancer, and *EGFR* mutations were most common in lung cancer.

**Figure 7:**
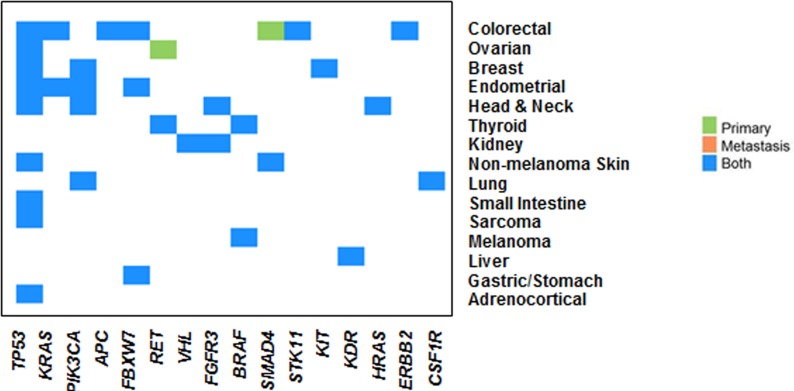
Concordance of mutations between matched primary and metastatic tumors 64 paired primary and metastatic pairs were examined; 46 pairs had somatic mutations, 24 pairs had actionable mutations. There is high concordance between primary and metastatic tumors, looking at both somatic and potentially actionable mutations. There was one colorectal patient who had a *SMAD4* mutation detected in the primary tumor that was not detected in the paired metastasis and one patient with fallopian tube tumor who had a *RET* mutation in the primary not detected in the metastasis and a *TP53* mutation in the metastasis that was not detected in the primary.

We then examined 64 paired primary and metastatic samples from this cohort; 46 pairs (72%) had somatic mutations detected on this platform; 24 pairs (52%) had actionable mutations. Analysis was undertaken to examine the concordance of mutations detected between paired primary and metastatic lesions (Figure 7). We found highly similar mutations in paired samples from primary and metastatic tumors, both when looking at all somatic mutations as well examining the subgroup of actionable mutations exclusively. There was one colorectal patient who had a *SMAD4* mutation detected in the primary tumor that was not detected in the paired metastasis and one patient with fallopian tube tumor who had a *RET* mutation in the primary (not detected in the metastasis) and a *TP53* mutation in the metastasis (not detected in the primary). When looking solely at potentially actionable mutations, there was concordance between all but one paired primary and metastatic samples (Figure 7). The high degree of concordance may reflect the depth of sequencing being able to detect mutations present in subclones or may alternatively reflect the limited heterogeneity in the hotspot mutations analyzed given the limitations of a 46 gene panel.

**Figure 8:**
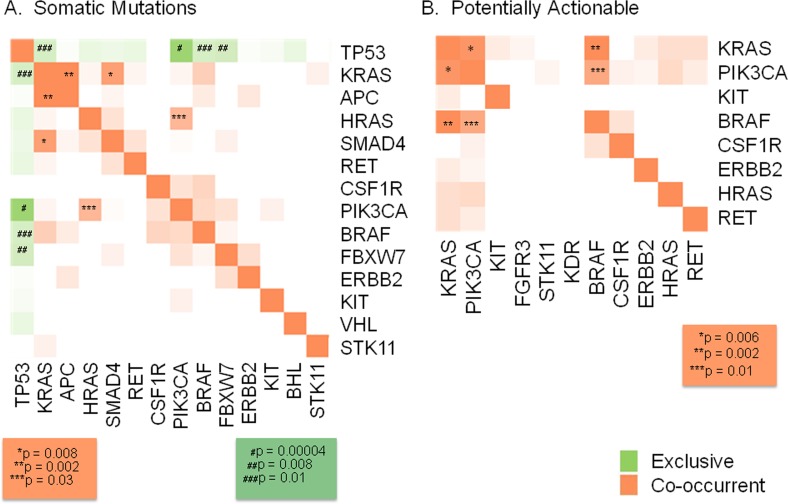
Mutual exclusivity and co-occurrence of mutations When examining the co-occurrence and exclusivity of various mutations the most notable findings were the mutual exclusivity between somatic mutations in *TP53* and *PIK3CA* (*p* = 0.00004)*, FBXW7* (*p* = 0.008), *BRAF and KRAS* (*p* = 0.01), and the co-occurrence of *KRAS* and *SMAD4* (*p* = 0.008), *KRAS* and *APC* (*p* = 0.002) and *HRAS* and *PIK3CA* (*p* = 0.03) (Figure 8a). Looking only at potentially actionable mutations, there was significant co-occurrence between *KRAS* and *PIK3CA* (*p* = 0.006), *KRAS* and *BRAF* (*p* = 0.002), and *BRAF* and *PIK3CA* (*p* = 0.01) (Figure 8b).

Finally, we examined the co-occurrence and exclusivity of various mutations (Figure 8). The most notable findings were the mutual exclusivity between somatic mutations in *TP53* and *PIK3CA* (*p* = 0.00004)*, FBXW7* (*p* = 0.008), *BRAF* and *KRAS* (*p* = 0.01), and the co-occurrence of somatic mutations in *KRAS* and *SMAD4* (*p* = 0.008), *KRAS* and *APC* (*p* = 0.002) and *HRAS* and *PIK3CA* (*p* = 0.03) (Figure 8A). Looking only at potentially actionable mutations, there was significant co-occurrence between *KRAS* and *PIK3CA* (*p* = 0.006), *KRAS* and *BRAF* (*p* = 0.002), and *BRAF* and *PIK3CA* (*p* = 0.01) (Figure 8B). It is unclear whether these events are solely driven by “mutual exclusivity” of the pathways involved or the potential that particular mutation events dominate specific lineages (i.e. PIK3CA mutations in luminal breast cancer and TP53 in basal breast cancer), but warrants further investigation.

## DISCUSSION

The current study has examined the landscape of mutations found in a heterogeneous population of patients with advanced cancer enrolled on a genomic profiling protocol at a dedicated cancer center. We analyzed the first 500 patients tested on a hotspot mutation panel enrolled through the Department of Investigational Cancer Therapeutics at MD Anderson, filtering out probable germline variants and looking more closely at somatic and actionable mutations found through hotspot profiling using the 46 gene panel. We were able to detect likely somatic mutations in over half of our patients (57%) and mutations in actionable genes in 30% of the overall population (Figure 1).

As noted, a gene was considered actionable if it could be directly or indirectly targeted with an approved or investigational therapy and there is some evidence of preclinical/clinical effectiveness in tumors harboring genetic alterations in the gene [9]. This definition is not tissue-specific and may not reflect therapeutic efficacy or clinical relevance across all cancer types. Furthermore, it does not reflect availability of trials for a specific patient which may be limited due to tumor site, slots on the trial or disqualifying criteria for trials such as performance status, prior therapy, brain metastasis or prior malignancy. It has been well-established that within the same gene, the type and site of mutation identified (with resultant differences in protein expression and function) may manifest with variable functional and biologic effects. Therefore, the definition of a gene as “actionable” is an oversimplification; not all mutations in that gene affect function and are “actionable”.

In our study *TP53* is not included in our classification of potentially actionable mutations, although there are studies utilizing *TP53* as a biomarker of therapeutic response, approaches to inhibit dominant-negative functions of mutant p53, and therapies to rescue p53 loss of function. In breast cancer, *TP53* mutations have been used as a marker of responsiveness to dose-dense epirubicin-cyclophosphamide [10] while in non-small cell lung carcinoma (NSCLC) it has been used as a marker of responsiveness to carboplatin/gemcitabine [11]. While the role of *TP53* mutations in chemo-naïve colorectal cancer patients is unclear [12-14], mutant *TP53* is a predictor of better clinical outcomes in patients with chemotherapy-refractory metastatic colorectal cancer treated with cetuximab [15]. This variability in mutational profiling findings amongst different diseases and in distinct clinical scenarios demonstrates the difficulty in classification schemata of this type. Additionally, there are current trials targeting either wild type or mutant *TP53* suggesting use as positive and negative selection strategy (i.e. inclusion or exclusion criteria respectively). However, due to the limited number of hotspots in *TP53* being targeted, we decided to consider *TP53* as not actionable at this time. In this analysis *IDH1/2* was also not considered actionable due to lack of trials addressing these alterations in solid tumors at the time of this study.

Similarly, in our analysis *KRAS* was classified as potentially actionable, despite its use in many studies as a negative predictor of EGFR-targeted therapy inhibitors [16] and being used as an exclusion criteria in a number of other trials. Our choice of inclusion of *KRAS* as an actionable target relates to the fact that there are early ongoing trials focused on downstream targeting of the MAPK pathway in *KRAS* mutant patients [17, 18], making it a potentially actionable target in some tumor types. Therefore, despite our current classification system, *KRAS* may not be actionable target, at least not simply targetable with single agent targeted therapy, in many diseases, including those in which the mutations occur in high proportion (e.g. pancreatic cancer) [19]. Therefore we displayed % of patients with mutations in actionable gene without *KRAS* in separately in Figure 4.

More work on these topics and curation of our findings is ongoing as clinical data accumulates. These examples demonstrate the complexity of profiling studies, particularly when examining mutational profiling across multiple cancers. Moving forward, cross-tumor comparison of mutational findings must include supporting and conflicting data to guide best-practice treatment decisions for patients. Other current limitations to studies of this type include the availability of slots on appropriate clinical trials even when data supporting matching of patients to trials of targeted therapies are available. This approach must account for the availability or lack of availability of appropriate trials within the institution. Finally, given the advanced nature of the disease in our patient population, there may be difficulties with patient eligibility for trials, even when trials are open and slots available. Therefore, the success of a program of this type will require continual data curation and iterative analysis, integration with disease-specific treatment teams and clinical trial groups, and an institutional commitment to multi-specialty management and application of a personalized medicine program.

Clearly the distribution of patients presented here is not representative of cancer patients at large. To avoid a referral bias, we focused this study to not all patients referred to the Department of Investigational Cancer Therapeutics (presuming patients with genomic alterations would be more likely referred) but rather those enrolled on the genomic profiling protocol in the Department It is notable that although our study population is limited to patients who had multiplex testing after being referred to the Department of Investigational Cancer Therapeutics, it is possible that selected patients already had single gene testing and had identified mutations (e.g. *BRAF* mutations), thus enriching for populations with actionable targets in certain diseases. There was certainly a referral bias impacting enrollment on this protocol and our data is not representative of the entire spectrum of patients seen in our Phase I program. The sample was enriched in patients with common mutations (e.g. BRAF-mutant melanoma), making the percentages of these patients higher than that which would be seen in the normal disease population. Not surprisingly, there were a high proportion of detected mutations in cancer types with known genetic alterations, such as colorectal cancer, pancreatic cancer, and melanoma given the selection of known actionable genes for use in the 46 gene panel. Additionally, the high proportion of mutations detected (greater than that which occurs in the baseline cancer population) may be related to pre-referral bias of patients selected for enrollment on this protocol. Diseases with well-characterized mutations were well-captured on this study (Figures 5 and 6, Table 1), demonstrating the utility of this streamlined approach in these particular patient populations. Less well-characterized cancers, such as sarcomas, were also well-represented in our patient population (*n* = 49) although the proportion of detected mutations with this panel were low (47% overall, 20% somatic, 6% actionable). This suggests that more extensive genomic profiling platforms, and in particular an ability to detect fusion genes, may have greater utility in patients with advanced sarcoma. Notably expanded mutation panels covering a larger number of actionable genes, the entire sequence of genes (especially tumor suppressor genes), as well as assays that give copy number variation is likely to lead to higher percentage of patients with actionable genomic alterations in each tumor type.

While successful discovery of known actionable targets for therapeutic selection is a high priority for studies of this type, we were also interested to examine the less common mutations identified through this molecular screening platform. While mutations in *KRAS*, *BRAF*, *PIK3CA*, and *EGFR* were found in cancer types expected, there were also several instances of actionable mutations identified in diseases for which these mutations have not been well characterized. For example, *HRAS* mutations were identified in a subset of patients with head and neck cancers, suggesting that use of downstream ERK or MEK inhibitors may be of benefit. Molecular screening techniques of this type when integrated into clinical decision-making systems may offer the opportunity for cross-cancer enrollment onto clinical trials and identification of patients who may benefit from targeted second or third-line therapies when standard-of-care regimens have failed.

Given the presence of paired samples from primary and metastatic sites within the same patients enrolled on protocol, we were also able to examine the concordance of identified mutations between primary and metastatic sites. We found a high concordance of mutations between the primary and temporally distant recurrent/metastatic sites, particularly when looking at actionable mutations. This suggests that archival tissue as well as newly biopsied samples may be appropriate for profiling and therapeutic selection in the majority of cases, expanding the cohort of patients who may be eligible for this type of profiling approach. However several studies, including our own previous studies, have shown that there may be genomic evolution with progression and selection pressure with targeted therapy [20-22]. Thus, repeat biopsy and profiling may be a considered as clinically indicated particularly when larger panels of aberrations are considered.

Finally, we examined the co-occurrence and mutual exclusivity of mutations, in order to address the concept of personalized profiling to inform decisions regarding the benefit (or potential lack of utility) of combinatorial therapies for specific patients with frequently occurring co-mutations. We found that within the samples with potentially actionable mutations, *PIK3CA* mutations and *KRAS* mutations were frequently co-occurring (*p* = 0.006), followed by *KRAS* and *BRAF* (*p* = 0.002), and *PIK3CA* and *BRAF* mutations (*p* = 0.01). The data presented here is generated from a small sample set and is not representative of each disease type, and thus should be considered hypothesis-generating only. However these results highlight a challenge to current genotype-selected single-agent trial strategies, as patients may have more than one therapeutic target or may have an actionable alteration in the presence of a resistance marker. Future studies are planned to examine these trends within disease-specific cohorts, where assessment of therapeutic approaches may be more similar and predictable and combination therapies may be more easily applied and evaluated.

Overall, the utilization of molecular profiling as a real-time tool for practicing clinicians has arrived. The current dilemma is to facilitate the translation of molecular findings into clinical decision-making in a feasible timeframe with appropriate decision-support. While whole exome or genome profiling is appealing in its breadth, focused sequencing approaches such as hotspot mutational profiling, can be accomplished in a more-realistic timeframe for use in day-to-day clinical care. We have demonstrated the feasibility of this approach in a heterogeneous patient population. Moving forward, we are integrating this data into treatment algorithms across cancer types with the goal of expanding the potential availability of targeted agents to the appropriately-selected patients for whom some benefit of therapies may be expected. Much work still needs to be done to focus the target selection to capture the greatest proportion of mutations across various disease types, to capture genomic alterations in rare cancer types, and to optimize the process to be accessible to clinicians rendering care on a routine basis. This will include simplification of molecular data presentation, automatic curation of germline/somatic/actionable mutation categories, determining the level of evidence for therapeutic implications of alterations in selected genes across multiple disease types, and building knowledge bases such as the websites “personalizedcancertherapy.org” (maintained by MD Anderson) and “mycancergenome” (initiated by Vanderbilt Cancer Center). Finally, it will require infrastructural foundations to build a pipeline of trials that leverage genomic testing and real-time processing and analytics to allow this type of data to be available, interpretable, and applicable to patients in a useful timeframe.

## MATERIALS AND METHODS

We reviewed data from the first 500 patients prospectively enrolled on the IRB-approved protocol in the in the Department of Investigational Cancer Therapeutics at MD Anderson. Hematoxylin and eosin stained tissue sections were reviewed, and tumor areas and tumor percentage cellularity was asssessed. Only specimens with > 20% tumor in the circled area were analyzed. Archival tumor DNA was tested in a Clinical Laboratory Improvement Amendments (CLIA) environment for 740 hotspot mutations in 46 genes (Suppl Table 1) (AmpliSeq Cancer Panel) using an Ion Torrent Personal Genome Machine Sequencer (Life Technologies, CA) [6]. This platform has already been extensively validated against orthogonal platforms [6-8]. Adequately covered amplicons were defined as those having total coverage depth of greater than or equal to 250 reads, or for which an orthogonal mutation analysis testing has been performed. For clinical purposes, we determined the effective lower limit of detection of this assay (analytical sensitivity) for single nucleotide variations to be in the range of 5% (one mutant allele in the background of nineteen wild type alleles) to 10% (one mutant allele in the background of nine wild type alleles) by taking into consideration the depth of coverage at a given base and the ability to confirm low level mutations using independent conventional platforms. Hot spot mutations as well as mutations detected in that region were reported. The data was analyzed using R Statistical Software (R Foundation for Statistical Computing, Vienna, Austria).

Detected mutations were identified as either likely germline variants or likely somatic based upon pre-established definitions (Suppl Table 2). Germline variants were defined by identifying and grouping variants based on relative prevalence within the MD Anderson patient population. The data was joined against dbSNP v.138 to pull in global minor allele frequency (GMAF) numbers where available. Mutations were labelled germline if GMAF was ≥ 0.001. Although *TP53* R273H had a GMAF ≥ 0.001, it was characterized as somatic due to additional supporting data. For outlier calls, variant frequency histograms for all patients at MD Anderson were examined to identify those demonstrating bimodal distribution, which would suggest hetero- and homozygous peaks consistent with germline variants. These histograms were created for all questionable outliers and germline calls in this group were made on a case-by-case basis. The designated somatic mutations were then filtered for mutations in 26 actionable genes (Suppl Table 3). Finally, these actionable mutations were analyzed for tissue-specific relevance as well as for cross-tissue comparison. Tissue-types with ≥ 10 patients were included for further analysis.

## SUPPLEMENTARY TABLES


