# Supplementary material for: Clinical next generation sequencing to identify actionable aberrations in a phase I program
Source: Oncotarget. 2015 May 8;6(24):20099–110. doi: 10.18632/oncotarget.4040 (PMC4652990; doi:10.18632/oncotarget.4040)
Supplement: Supplementary file 1 [file oncotarget-06-20099-s001.pdf]

Clinical next generation sequencing to identify actionable aberrations in a phase I program

Supplementary Material

Supplementary Table 1: Codons tested on Ampliseq 46 gene Assay.

Gene	Exons (codons) tested
**ABL1**	4 (237-260), 5 (275-283), 6 (303-319), 7 (387-412)
**AKT1**	3 (16-59)
**ALK**	23 (1172-1177), 25 (1259-1277)
**APC**	16 (865-886), 16 (1105-1122), 16 (1289-1322), 16 (1349-1382), 16 (1430-1467), 16 (1487-1509), 16 (1549-1564)
**ATM**	8 (343-355), 9 (395-412), 12 (601-614), 17 (837-862), 26 (1307-1324), 34 (1674-1693), 35 (1733-1758), 36 (1785-1802), 39 (1935-1957), 50 (2436-2445), 54 (2650-2667), 55 (2693-2715), 56 (2721-2739), 59 (2888-2891), 61 (2937-2950), 63 (2996-3016), 63 (3037-3052)
**BRAF**	11 (439-471), 15 (581-605)
**CDH1**	3 (69-92), 8 (351-373), 9 (395-415)
**CDKN2A**	2 (51-76)
**CSF1R**	7 (299-318), 22 (952-973)
**CTNNB1**	3 (12-45)
**EGFR**	3 (89-125), 7 (280-297), 15 (575-601), 18 (698-722), 19 (729-761), 20 (766-790), 20 (803-823), 21 (830-866)
**ERBB2**	19 (753-769), 20 (772-797), 21 (832-852), 21 (875-883)
**ERBB4**	3 (136-141), 4 (177-186), 6 (234-247), 7 (272-289), 8 (303-322), 9 (343-363), 15 (588-619), 23 (923-943)
**FBXW7**	5 (264-279), 8 (381-400), 9 (450-472), 10 (478-506), 11 (566-583)
**FGFR1**	4 (121-139), 7 (247-268)
**FGFR2**	7 (250-268), 7 (297-313), 9 (367-395), 12 (546-558)
**FGFR3**	7 (247-268), 9 (377-409), 14 (634-653), 16 (681-712), 18 (790-807)
**FLT3**	11 (441-458), 14 (569-575), 16 (662-682), 20 (828-846)
**GNAS**	8 (196-218)
**HNF1A**	3 (198-217), 4 (253-282)
**HRAS**	2 (5-23), 3 (48-79)
**IDH1**	4 (118-134)
**JAK2**	14 (604-622)
**JAK3**	13 (568-578), 16 (709-729)
**KDR**	6 (240-258), 7 (267-280), 11 (472-490), 19 (872-892), 21 (959-985), 26 (1138-1161), 27 (1192-1216), 30 (1301-1321), 30 (1336-1356)
**KIT**	2 (47-69), 9 (501-514), 10 (536-549), 11 (550-585), 13 (641-664), 14 (664-684), 15 (714-728), 17 (807-828), 18 (836-854)
**KRAS**	2 (5-28), 3 (40-67), 4 (136-150)
**MET**	2 (160-187), 2 (362-379), 14 (992-1017), 16 (1105-1126), 19 (1247-1268)
**MLH1**	12 (373-393)
**MPL**	10 (499-522)
**NOTCH1**	26 (1566-1605), 27 (1673-1697)
**NPM1**	11 (283-295)
**NRAS**	2 (6-22), 3 (53-69)
**PDGFRA**	12 (552-570), 14 (647-668), 15 (668-688), 18 (819-847)
**PIK3CA**	2 (77-98), 5 (328-351), 8 (418-422), 10 (533-551), 14 (688-716), 21 (1019-1049), 21 (1065-1069)
**PTEN**	1 (5-24), 3 (55-70), 6 (167-184), 7 (212-222), 7 (240-266), 8 (282-300), 8 (316-342)
**PTPN11**	3 (53-82), 13 (486-506)
**RB1**	4 (132-154), 6 (195-203), 11 (350-371), 17 (549-565), 18 (566-585), 20 (655-680), 21 (703-724), 22 (743-770)
**RET**	10 (609-627), 11 (630-654), 13 (762-774), 15 (880-901), 16 (914-931)
**SMAD4**	3 (109-128), 5 (167-184), 6 (228-247), 8 (304-319), 9 (330-363), 10 (385-404), 11 (444-472), 12 (497-526)
**SMARCB1**	2 (39-55), 4 (154-167), 5 (182-203), 9 (373-386)
**SMO**	3 (186-218), 5 (310-340), 6 (399-418), 9 (516-542), 11 (626-646)
**SRC**	14 (514-534)
**STK11**	1 (30-62), 4 (174-1999), 6 (253-281), 8 (325-360)
**TP53**	2 (1-18), 4 (81-114), 5 (126-135), 5 (149-181), 6 (187-223), 7 (230-253), 8 (269-306), 10 (332-344)
**VHL**	1 (88-110), 2 (120-149), 3 (157-175)

Supplementary Table 2: List of likely germline variants

Gene	Codon	WildType	VariantType
**ABL1**	247	K	R
**APC**	870	P	S
**APC**	1317	E	Q
**ATM**	410	V	A
**ATM**	604	P	S
**ATM**	858	F	L
**ATM**	1309	A	T
**ATM**	1691	S	R
**FGFR3**	384	F	L
**JAK3**	132	P	T
**JAK3**	722	V	I
**KDR**	482	C	R
**KDR**	1356	V	A
**KIT**	541	M	L
**MET**	168	E	D
**MET**	362	M	T
**MET**	375	N	S
**MET**	1010	T	I
**MLH1**	384	V	D
**PIK3CA**	391	I	M
**STK11**	354	F	L
**TP53**	273	R	H

Supplementary Table 3: List of potentially actionable genes and therapeutic implications

Gene	Potential Therapeutic Implications
**AKT1**	Treatment with AKT or mTOR inhibitors
**ATM**	Treatment with PARP inhibitors
**BRAF**	Treatment with BRAF inhibitors
**CDKN2A**	Treatment with CDK 4/6 inhibitors
**CSF1R**	Treament with CSF1R inhibitors
**EGFR**	Treatment with EGFR inhibitors
**ERBB2 (HER2)**	Treatment with HER2 inhibitors, monoclonal antibodies, and targeted vaccines
**ERBB4 (HER4)**	Treatment with HER4 inhibitors
**FGFR1**	Treatment with FGFR1 inhibitors
**FGFR2**	Treatment with FGFR2 inhibitors
**FGFR3**	Treatment with FGFR3 inhibitors
**GNA11**	Treatment with PKC and MEK inhibitors
**HRAS**	Treatment with MEK Inhibitors
**JAK2**	Treatment with JAK inhibitors
**JAK3**	Treatment with JAK inhibitors
**KDR**	Treatment with KDR inhibitors
**KIT**	Treatment with KIT inhibitors
**KRAS**	Treatment with MEK Inhibitors
**MET**	Treatment with MET inhibitors
**NOTCH1**	Treatment with Gamma Secretase inhibitors (GSIs)
**NRAS**	Treatment with MEK inhibitors
**PDGFRA**	Treatment with PDGFRA inhibitors
**PIK3CA**	Treatment with PI3K, AKT, or mTOR inhibitors
**PTEN**	Treatment with p110beta, AKT, or mTOR inhibitors
**RET**	Treatment with RET inhibitors
**STK11**	Treatment with mTOR or AMPK inhibitors
